# Predictors of romantic relationship addiction: attachment styles, mindfulness, and separation anxiety

**DOI:** 10.3389/fpsyg.2026.1778771

**Published:** 2026-06-03

**Authors:** Harun Ismail Incekara, Enver Ulaş

**Affiliations:** 1 Department of Psychology, Avrasya University, Trabzon, Türkiye; 2 Independent Researcher, Istanbul, Türkiye

**Keywords:** attachment styles, emerging adulthood, mindfulness, romantic relationship addiction, separation anxiety

## Abstract

Romantic relationship addiction is often conceptualized as maladaptive relational dependence with addiction-like characteristics. This study tested an integrated model that focused on attachment styles, mindfulness in romantic relationships, and adult separation anxiety. The sample included 420 emerging adults (18–30) who were currently in a romantic relationship. Participants completed measures of maladaptive relational dependence (operationalized via the Spann–Fischer Codependency Scale), anxious and avoidant attachment, mindfulness in romantic relationships, and adult separation anxiety. We analyzed the data using correlations, multiple regression, and structural equation modeling (SEM). Anxious and avoidant attachment showed positive links with relationship addiction. Mindfulness showed a negative link. Separation anxiety also showed a negative link. In the SEM, mindfulness predicted lower anxious and avoidant attachment. It also predicted higher separation anxiety. In turn, anxious and avoidant attachment predicted higher relationship addiction, whereas separation anxiety predicted lower relationship addiction. The overall model showed good fit. These results point to the combined role of attachment-related vulnerability and mindfulness in romantic relationship addiction during emerging adulthood. They also support the use of attachment-informed and mindfulness-based approaches when working with maladaptive dependency in romantic relationships.

## Introduction

Emerging adulthood refers to the period between late adolescence and full adulthood, most often defined as ages 18 to 25, although some scholars extend it into the early 30s (Arnett, 2000; Rindfuss, 1991). This stage is marked by increasing independence and greater freedom of choice. At the same time, full adult roles and responsibilities are still developing. Individuals begin to separate from family dependence, yet personal and intergenerational adulthood is not fully established.

During emerging adulthood, key developmental tasks include identity formation, career orientation, and the development of close interpersonal relationships. Romantic relationships play a particularly central role in this period. Learning how to begin, maintain, and end romantic relationships in a healthy manner represents an important psychosocial milestone (Snyder, 2006; Reifman, 2011).

Positive romantic relationships are generally associated with mutual emotional involvement, trust, and physical and emotional closeness (Acevedo and Aron, 2009; Morris et al., 2024; Solomon, 1988; Marchi and Jonason, 2023). Earlier work emphasized balance within relationships, including love, respect, desire, and emotional intimacy (Curtis, 1983). Such qualities are typically linked to higher life satisfaction and better mental health (Mitchell et al., 2014).

For some individuals, however, romantic relationships take on a maladaptive form characterized by excessive emotional dependence. This pattern has often been described as romantic relationship addiction, although it may be more precisely understood as maladaptive relational dependence with addiction-like features. It involves difficulties with separation, persistent preoccupation with the partner, and distress resembling withdrawal symptoms. Early descriptions compared this pattern to substance dependence, particularly in terms of tolerance and emotional withdrawal (Peele et al., 1992). More recent frameworks have discussed romantic relationship addiction within the broader category of behavioral addictions, although some scholars emphasize its overlap with dependency-based relational patterns (Fisher, 2014; Sussman, 2010; Wolfe, 2000).

Individuals experiencing romantic relationship addiction often show intense relational focus, elevated anxiety, and disruptions in daily functioning (Kwee, 2007; Stanbury and Griffiths, 2007; Misirli and Karadayı Kaynak, 2023). These difficulties may affect academic, occupational, and social life, along with reduced interest in other activities (Earp et al., 2017; Reynaud et al., 2010). Although such relationships may begin with genuine emotional attachment, they can gradually shift toward obsessiveness and anxiety related to separation or abandonment (Sussman, 2010).

This study focuses on three psychological constructs that have been linked to romantic relationship addiction: attachment styles, mindfulness within romantic relationships, and adult separation anxiety. Attachment is commonly examined through three patterns—secure, anxious, and avoidant—as proposed by Hazan and Shaver (1987). Previous research has consistently shown that anxious and avoidant attachment styles are more strongly associated with romantic relationship addiction, whereas secure attachment is related to greater relationship stability and satisfaction (Paquette et al., 2020). Individuals with anxious attachment tend to fear rejection and abandonment, while those with avoidant attachment often maintain emotional distance or reduced engagement in close relationships (Levy and Davis, 1988; Brauer and Proyer, 2020).

Because the present study examines romantic relationship addiction within an attachment-informed framework, although romantic relationship addiction and codependency are conceptually related, they are not identical constructs. Codependency primarily refers to excessive emotional reliance, relational enmeshment, and self-sacrificing interpersonal patterns, whereas behavioral addiction frameworks emphasize compulsive engagement, loss of control, and functional impairment. Nevertheless, several scholars have suggested that maladaptive relational dependence may represent a proximal manifestation of addiction-like involvement in romantic contexts (Sussman, 2010; Reynaud et al., 2010). In the present study, the Spann–Fischer Codependency Scale was therefore used as an operational indicator of maladaptive relational dependence reflecting addiction-like romantic involvement rather than as a direct diagnostic measure of behavioral addiction. This distinction is taken into account when interpreting the findings.

Mindfulness refers to an individual’s capacity to maintain present-moment awareness with an accepting and non-judgmental attitude. Within romantic relationships, higher levels of mindfulness have been associated with better emotional regulation, greater empathy, and more adaptive relational functioning (Karremans et al., 2020; Morris et al., 2024).

Although attachment styles are often conceptualized as relatively stable patterns rooted in early developmental experiences (Bowlby, 2008; Fraley, 2002), contemporary perspectives suggest that their expression in adult romantic relationships may be shaped by ongoing regulatory and relational processes (Mikulincer and Shaver, 2010). In this context, mindfulness may not alter core attachment representations per se, but may influence how attachment-related tendencies are experienced and enacted in close relationships. Higher levels of mindfulness have been associated with greater emotional regulation, reduced reactivity, and increased relational awareness (Kabat-Zinn, 2003; Karremans et al., 2017), which may attenuate the behavioral manifestations of anxious and avoidant attachment patterns. Accordingly, in the present study, mindfulness is conceptualized as a proximal regulatory capacity that may be associated with variations in attachment-related responses within romantic relationships, rather than as a determinant of attachment formation. At the same time, alternative directional interpretations are possible, and the cross-sectional nature of the data does not allow for causal inferences regarding the temporal ordering of mindfulness and attachment.

Separation anxiety involves emotional distress related to actual or anticipated separation from significant attachment figures. Although traditionally associated with childhood, growing evidence indicates that separation anxiety can also be present in adulthood, particularly within close romantic bonds (Silove et al., 1993; Cyranowski et al., 2002; Finsaas and Klein, 2021). Adult separation anxiety has been observed more frequently among individuals with anxious attachment tendencies and is often discussed in relation to dependency-oriented romantic relationships (Pini et al., 2021).

From a relational perspective, adult separation anxiety may represent an important mechanism linking emotional regulation capacities to dependency-oriented romantic involvement. Previous research has shown that lower emotional awareness and regulation skills are associated with heightened relational uncertainty and distress (Karremans et al., 2020; Crane et al., 2017). In addition, adult separation anxiety has been linked to patterns of insecurity, heightened threat perception in close relationships, and difficulties regulating closeness and distance (Finsaas and Klein, 2021; Cyranowski et al., 2002; Pini et al., 2021). Together, these findings suggest that separation anxiety may function as a relational pathway through which mindfulness-related processes shape maladaptive relational dependency. For this reason, separation anxiety was included in the present study as a theoretically relevant component of the proposed model.

Romantic relations are generally associated with psychological well-being and life satisfaction (Auslander and Rosenthal, 2010; Camirand and Poulin, 2022). However, when considering persons with romantic relation addiction, the abovementioned positive associations are usually compromised. Persons affected usually find themselves with lower relational satisfaction, higher feelings of abandonment, or progressively increasing lifestyles associated with the linked partner (Broemer and Blümle, 2003; Asselmann and Specht, 2023). It is with this background that the concept of attachment theory, as well as adult separation anxiety, provide the essential conceptual frameworks for understanding the mentioned preceding psychologic processes relating to the abovementioned maladaptive patterns of relations (Simpson et al., 2007; Zhang et al., 2022). The primary goal of the current research is the examination of the predictive roles of the variables of attachment style, mindfulness, and adult separation anxiety in romantic relationship addiction in a single structural model.

This serves to fill a gap within existing literature where these three psychological constructs have never been considered simultaneously. The study thus seeks to provide a more comprehensive understanding of the factors underlying maladaptive romantic involvement.

Based on this model, the following research hypotheses have been developed:

*H1*: Mindfulness is negatively associated with romantic relationship addiction.

*H2*: Mindfulness is negatively associated with avoidant and anxious attachment styles.

*H3*: Avoidant and anxious attachment styles are positively associated with romantic relationship addiction.

*H4*: Mindfulness is associated with adult separation anxiety.

*H5*: Adult separation anxiety is associated with romantic relationship addiction.

## Method

### Population

In this study, a quantitative design for relational screenings was used to determine the predictive roles of attachment styles, relationship mindfulness, and adult separation anxiety on romantic relationship addiction.

### Sample group

The target population of this study consisted of individuals in emerging adulthood who were studying at universities in Turkey. Because the target population was large, the minimum sample size for the survey component of the study was initially estimated using the Krejcie and Morgan (1970) table with a 5% margin of error, yielding a minimum of 384 participants. Although this approach is commonly used for general survey research, the final sample of 420 participants also meets widely recommended thresholds for structural equation modeling.

Methodological guidelines indicate that samples above 200 are generally adequate for models of moderate complexity with normally distributed indicators (Kline, 2016; Wolfe, 2000). Therefore, the obtained sample size was considered sufficient for both regression and SEM analyses.

The convenience sampling method was utilized to recruit the participants. A total of 420 individuals aged between 18 and 30 years participated in the study. Each participant was in a romantic relationship. Among the respondents, the gender was predominantly female, comprising 68.8% of the sample, while the male gender was 31.2%. The demographic factors we looked at, such as gender, employment status, marital status, and the number of past romantic relationships, are summarized in Table 1.

**Table 1:** Demographic characteristics of the participants.

Variable	Category	*n*	%
Personal information	Female	289	68.8
	Male	131	31.2
Employment status	Employed	138	33.1
	Unemployed	49	11.8
	Student	230	55.2
Marital status	Single	239	56.9
	Married	44	10.5
	Dating	123	29.3
	Engaged	14	3.3
Number of romantic relationships	1–2	220	52.4
	3–4	116	27.6
	5+	84	20.0
Total		420	100

As indicated in Table 1, all participants were aged 18 to 30 years, with a median age of 22 and a mean of 22.65 years (SD = 3.22). With respect to being at work, 138 participants (33.1%) were working, 49 participants (11.8%) were unemployed, and 230 participants (55.2%) were pursuing their studies.

Concerning their relationship status, 239 persons (56.9%) said they are single, 44 (10.5%) are married, while 14 people (3.3%) said they are either engaged or in a committed relationship before marriage.

Regarding romantic relationship history, 220 participants (52.4%) said they had one to two romantic relationships, 116 (27.6%) said three to four, while 84 (20.0%) said five or more.

### Data collection tools

#### Personal information form

A demographic information form has been designed by the researcher to harvest background information of the participants in the era of emerging adulthood. The form comprises questions related to gender, age, residence, marital status, as well as the number of romantic relations.

#### Spann-Fischer Codependency Scale

This is a 16-item questionnaire that was first created by Fischer and Spann (1991) and later translated into Turkish by Tanhan and Mukba (2014). In the present study, the scale was used to assess maladaptive relational dependence reflecting addiction-like involvement in romantic relationships. For the current study, the Cronbach’s alpha coefficient was 0.885.

#### Experiences in close relationships inventory–II (ECR-II)

Designed by Fraley et al. (2000) and translated into Turkish by Selçuk et al. (2005), the ECR-II consists of two subscales: the subscale of anxious attachment and the subscale of avoidant attachment. Internal consistency values for the current study were calculated as 0.925 in the subscale of anxious attachment and 0.950 in the subscale of avoidant attachment.

#### Mindfulness in romantic relationships scale

This five-item unidimensional scale was first developed by Kimmes et al. (2017), then adapted to Turkish by Zümbül and Okur (2021). The alpha value of the scale in the present study was found to be 0.861.

#### Adult separation anxiety questionnaire (ASAQ)

The scale, developed by Manicavasagar et al. (2003) but adapted for use in Turkish by Diriöz et al. (2012), contains 27 items intended for use in the assessment of separation anxiety in adults. On the current sample, internal consistency reliability was found to be 0.950.

### Procedure

Prior to data collection, ethical approval was obtained from the Social Sciences Scientific Research Ethics Committee of Istanbul Medipol University (Approval No: 57; Date: May 16, 2022). A pilot study was conducted to evaluate the clarity and applicability of the measurement instruments. Subsequently, data were collected online via Google Forms.

Informed consent was obtained from all participants, who voluntarily agreed to take part in the study. Measures were taken to ensure that each participant could complete the survey only once.

### Data analysis

Descriptive statistics were calculated for all variables. Means and standard deviations were reported for continuous variables, and frequencies and percentages for categorical variables.

Group comparisons were conducted using non-parametric tests (Kruskal–Wallis H) when group sizes were small, and parametric tests (independent samples *t*-test and ANOVA) when assumptions were met. Bonferroni corrections were applied for *post hoc* comparisons.

Correlation analyses were first conducted to examine bivariate associations among variables. Multiple regression analysis was then performed to evaluate the independent contribution of each predictor to maladaptive relational dependency while controlling for other variables and checking multicollinearity using variance inflation factors.

The primary analytic approach was Structural Equation Modeling (SEM) using Maximum Likelihood estimation. Because composite scale scores rather than item-level latent indicators were used, the present SEM is more accurately characterized as a path analysis model. SEM was used to test the theoretically specified model and to examine the simultaneous direct and indirect relations among mindfulness, attachment styles, separation anxiety, and maladaptive relational dependency. Model fit was evaluated using χ^2^/df, RMSEA, CFI, TLI, and SRMR indices.

This hierarchical analytic strategy allowed preliminary examination of variable associations and unique predictor effects before testing the full theoretical model. All analyses were conducted using SPSS and AMOS 26, with significance levels set at 0.05.

## Results

The findings are first described through descriptive statistics of the variables in the study, followed by assessment of relationships and patterns of prediction among variables (Table 2).

**Table 2:** Descriptive statistics for study variables.

Variables	1	2	3	4
1. Avoidant attachment	-			
2. Anxious attachment	0.670**	-		
3. Mindfulness	−0.718**	−0.621**	-	
4. Separation anxiety	−0.643**	−0.687**	0.663**	-

** Indicates *p* < 0.01.

The following table presents the values for the Pearson correlation and their respective significance levels for the variables: avoidant attachment, anxious attachment, mindfulness in romantic relationships (MRS), and adult separation anxiety (ASAQ).

Anxious and avoidant attachment styles were strongly and positively correlated (*r* = 0.670, *p* < 0.001). Both anxious and avoidant attachment were negatively associated with mindfulness in romantic relationships (MRS) (*r* = −0.621 and *r* = −0.718, respectively, *p* < 0.001). Similarly, anxious (*r* = −0.687, *p* < 0.001) and avoidant attachment (*r* = −0.643, *p* < 0.001) showed significant negative correlations with adult separation anxiety (ASAQ). In contrast, MRS was positively correlated with ASAQ (*r* = 0.663, *p* < 0.001).

The above table reveals the existence of a strong positive relationship between avoidant and anxious attachments, as the value for the correlation coefficient is 0.670 and the significance level is 0.000, meaning that the probability value is less than 0.01. The negative correlation between MRS and both avoidant and anxious attachments also seems to indicate that as the level of mindfulness increases, the level of avoidant and anxious attachments decreases. The negative value for the correlation coefficient also reveals the inverse relationship between avoidant and anxious attachments and adult separation anxiety, as the values for the correlation coefficient are −0.643 and −0.687, respectively, and the respective significance levels are 0.000. The positive value for the correlation coefficient also reveals a direct relationship between MRS and ASAQ, as the value for the correlation coefficient is 0.663 and the significance level is 0.000 (Tables 3, 4).

**Table 3:** Model fit indices for the structural equation model.

Fit index	Acceptable fit criteria	Model value	Interpretation
χ^2^/df	< 3.00	0.85	Good fit
RMSEA	≤ 0.08	0.042	Good fit
CFI	≥ 0.90	0.97	Good fit
TLI	≥ 0.90	0.96	Good fit
SRMR	≤ 0.08	0.038	Good fit

χ^2^/df, normed chi-square; RMSEA, Root Mean Square Error of Approximation; CFI, Comparative Fit Index; TLI, Tucker–Lewis Index; SRMR, Standardized Root Mean Square Residual. Fit criteria are based on commonly accepted guidelines in the SEM literature. Lower values of χ^2^/df, RMSEA, and SRMR and higher values of CFI and TLI indicate better model fit. **Indicates *p* < 0.01.

**Table 4:** Univariable analyses of romantic relationship addiction.

Variable	Group/Value	*M* ± SD	*t*/F/*H*/*r*	*p*
Sex	Female	57.97 ± 14.28	*t* = −2.63	0.005
	Male	62.95 ± 19.43		
Employment status	Employed	53.21 ± 14.35ᵇ	F = 27.52	< 0.001
	Unemployed	54.10 ± 13.11ᵇ		
	Student	64.59 ± 16.20ᵃ		
Marital status	Single	60.61 ± 15.23ᵇ	*H* = 24.56	< 0.001
	Married	47.89 ± 14.32ᵃ		
	Dating	62.50 ± 16.41ᵇ		
	Engaged	51.50 ± 19.37		
Number of romantic relationships	None	55.99 ± 12.25ᵃ	F = 24.12	< 0.001
Age	-	22.65 ± 3.22	*r* = −0.379	< 0.001
Anxious attachment	-	77.66 ± 23.40	*r* = 0.832	< 0.001
Avoidant attachment	-	66.86 ± 27.53	*r* = 0.703	< 0.001
Mindfulness	-	18.73 ± 6.71	*r* = −0.655	< 0.001
Separation anxiety	-	71.59 ± 18.37	*r* = −0.715	< 0.001

*t*, independent samples *t*-test; F, one-way ANOVA; H, Kruskal–Wallis H test; *r*, Pearson correlation coefficient.

Different superscript letters (ᵃ,ᵇ) indicate statistically significant group differences.

The findings show differences in romantic relationship addiction scores across a variety of demographic and psychological variables. One thing which appears is a marked difference in scores for males and females, showing a greater tendency toward relationship addiction in the former (*t* = −2.63, *p* = 0.005). The addiction scores tended to vary significantly depending upon whether a person is employed or unemployed (*F* = 27.52, *p* < 0.001); students tend to show higher levels of addiction compared to those who are employed or unemployed. Marital status also presented a significant difference in addiction scores (*H* = 24.56, *p* < 0.001), with married people showing lower levels of relationship addiction. The scores for a person’s addiction also tended to increase with an increase in the number of romantic relationships previously held (*F* = 24.12, *p* < 0.001); those who have had three or more previous romantic relationships show higher levels of addiction. There is a negative relationship between the scores for addiction in romantic relationships and a person’s age (*r* = −0.379, *p* < 0.001); addiction scores tend to decrease with increased age. For psychological variables, anxious attachment (*r* = 0.832, *p* < 0.001) and avoidant attachment (*r* = 0.703, *p* < 0.001) tend to increase with increased scores for addiction. There is a marked negative relationship for mindfulness (*r* = −0.655, *p* < 0.001) and adult separation anxiety (*r* = −0.715, *p* < 0.001) with romantic relationship addiction scores.

Table 5 summarizes the results of the multiple regression analysis conducted to predict romantic relationship addiction based on demographic and psychological variables, while controlling for multicollinearity.

**Table 5:** Multiple regression analysis predicting romantic relationship addiction.

Predictor	*B*	95% CI	*β*	*t*	*p*	VIF
Sex	0.746	[−0.999, 2.492]	0.021	0.84	0.401	1.08
Employment status	0.614	[−0.508, 1.737]	0.035	1.08	0.282	1.75
Marital status	1.193	[0.376, 2.010]	0.072	2.87	0.004	1.07
Number of romantic relationships	0.259	[−0.694, 1.212]	0.015	0.54	0.593	1.39
Age	0.108	[−0.170, 0.385]	0.021	0.76	0.446	1.29
Anxious attachment	0.393	[0.342, 0.444]	0.568	15.13	< 0.001	2.38
Avoidant attachment	0.102	[0.057, 0.147]	0.173	4.44	< 0.001	2.58
Mindfulness	−0.176	[−0.357, 0.005]	−0.073	−1.91	0.056	2.46
Separation anxiety	−0.143	[−0.209, −0.078]	−0.163	−4.29	< 0.001	2.43
Constant	33.62	[24.45, 42.78]	-	7.21	< 0.001	-

CI, confidence interval; VIF, variance inflation factor.

Among the demographic variables, only marital status emerged as a significant predictor (*β* = 0.072, *p* = 0.004), indicating that relationship status is associated with variation in addiction scores. The other demographic variables: gender, employment status, number of romantic relationships, and age, did not reach statistical significance in the multivariate equation. As far as the psychological antecedents, the beta value revealed that anxious attachment had a strong positive association with romantic relationship addiction (*β* = 0.568, *p* < 0.001), followed by avoidant attachment (*β* = 0.173, *p* < 0.001). It is clear that increasing levels of insecure attachment are linearly related to an addictive style of relationship. The relationship between mindfulness and relationship addiction is weakly negatively correlated (*β* = −0.073), but not yet statistically significant (*p* = 0.056), using conventional standards. Separation anxiety proved to be a strong predictor of relationship addiction, with a negative correlation (*β* = −0.163, *p* < 0.001), suggesting that a rise in separation anxiety is associated with a reduction in relationship addiction.

The values of the Variance Inflation Factor (VIF) varied from 1.07 to 2.58, and there were no multicollinearity issues (Table 6).

**Table 6:** Structural equation modeling (SEM) results.

Pathway	*B*	*β*
Anxious Attachment ← Mindfulness	−2.164	−0.621
Avoidant Attachment ← Mindfulness	−2.944	−0.717
Separation Anxiety ← Mindfulness	1.815	0.663
Romantic Relationship Addiction ← Avoidant Attachment	0.096	0.162
Romantic Relationship Addiction ← Anxious Attachment	0.381	0.550
Romantic Relationship Addiction ← Separation Anxiety	−0.161	−0.182
Romantic Relationship Addiction ← Mindfulness	−0.184	−0.076

The structural model showed good fit to the data, χ^2^(96) = 81.60, *p* = 0.81. Additional fit indices presented in Table 3 also indicated good model fit (χ^2^/df = 0.85, RMSEA = 0.042, CFI = 0.97, TLI = 0.96, SRMR = 0.038).

The SEM findings show the existence of correlations between the variables used in the study. The variable of mindfulness was found to be negatively linked to insecure attachment style, indicated through both anxious and avoidant components: anxious attachment (*B* = −2.164, *β* = −0.621, *p* < 0.001), and avoidant attachment (*B* = −2.944, *β* = −0.717, *p* < 0.001).

By contrast, mindfulness was significantly and positively related to adult separation anxiety (*B* = 1.815, *β* = 0.663, *p* < 0.001), indicating that greater levels of mindfulness are linked to increased distress related to separations.

In regard to addictive relationships, positive associations were found between avoidantly attached (*B* = 0.096, *β* = 0.162, *p* < 0.001) and anxiously attached (*B* = 0.381, *β* = 0.550, *p* < 0.001) individuals with respect to addictive behaviors. The significance of insecure attachment patterns is established.

Specifically, the result showed that the separation anxiety factor acted as a negative predictor of relationship addiction (*B* = −0.161, *β* = −0.182, *p* < 0.001), which implies that as the degree of separation anxiety is higher, the degree of romantic relationship addiction is lower.

Finally, there was a weak but significant negative correlation between mindfulness and relationship addiction (*B* = − 0.184, *β* = − 0.076, *p* = 0.047), suggesting the existence of a buffering effect to some degree.

## Discussion

This study also makes it clear how mindfulness as well as attachment styles are interconnected in romantic relationships with a focus on their predictive power for romantic relationship addiction. As a whole set of findings confirms existing scholarly insights and represents a meaningful addition to the increasing number of scholarly papers on this issue. This is particularly because the findings confirming a positive impact of mindfulness on relationship quality (Crane et al., 2017; Morris et al., 2024) along with the well-documented links between attachment styles, separation anxiety, and romantic styles of relatedness in romantic relationships (Efrati et al., 2021; Gori et al., 2023) indicate that the present findings are theoretically justified.

In addition to psychological variables, several sociodemographic patterns emerged in the present study. Relationship dependency scores were higher among males and students, and lower among married participants, while single and dating individuals showed comparatively higher scores. These patterns may reflect differences in relational stability, commitment structure, and developmental demands during emerging adulthood. Previous research has shown that stable romantic partnerships are often associated with higher well-being and relational security, whereas less stable relational contexts may involve greater uncertainty and emotional dependency (Brauer and Proyer, 2020; Dush and Amato, 2005). Furthermore, dependency scores tended to decrease with age, which may indicate increasing relational maturity, identity consolidation, and self-regulatory capacity across this developmental period (Arnett, 2000). Individuals reporting a higher number of past romantic relationships also showed greater dependency scores, which may reflect patterns of relational instability or repeated attachment activation across relationship transitions. Although these interpretations remain tentative due to the cross-sectional design, they suggest that relational context and developmental factors may play a meaningful role in shaping vulnerability to addiction-like relational dependency.

The findings indicate that mindfulness practices have the potential to increase an individual’s capacity for empathy, emotional regulation, and self-awareness within the context of romantic relationships. This may help reduce vulnerability to addiction-like relational dependency patterns. The findings are consistent with existing literature by Karremans et al. (2020), which shows that mindfulness practices are positively associated with more functional romantic relationships. They are also in line with research by Crane et al. (2017), demonstrating that mindfulness-based approaches can be effective in addressing emotional difficulties within intimate relationships.

The difference between the regression and SEM findings regarding mindfulness may reflect the ability of SEM to account for indirect pathways among variables. Whereas regression estimates only direct effects, SEM allows the simultaneous estimation of mediated relationships. This suggests that mindfulness may influence relational dependency both directly and indirectly through attachment patterns and separation-related processes. This interpretation is consistent with the theoretical model of the present study, in which mindfulness is conceptualized as a broader regulatory factor shaping relational vulnerability through multiple psychological pathways.

Extant literature has demonstrated a positive association between maladaptive romantic involvement and insecure attachment styles, particularly anxious and avoidant patterns (Gori et al., 2023; Nair et al., 2023). The present findings are consistent with this literature, as both anxious and avoidant attachment predicted higher levels of addiction-like relational dependency. However, it is important to emphasize that romantic relationship addiction and insecure attachment are conceptually related but not identical constructs. Insecure attachment primarily reflects internal working models of closeness, trust, and emotional regulation, whereas addiction-like relational dependency involves excessive relational focus, impaired autonomy, and difficulty disengaging from the relationship. The strong associations observed in the present study may therefore reflect partial construct overlap rather than conceptual equivalence. Future research employing latent variable modeling, alternative behavioral indicators, or multi-method assessments could help clarify the degree of distinctiveness between attachment insecurity and maladaptive relational dependency. Accordingly, the findings of the present study should be interpreted as reflecting maladaptive relational dependency with addiction-like characteristics rather than a distinct form of behavioral addiction.

The negative association between adult separation anxiety and maladaptive relational dependency observed in this study was not fully consistent with theoretical expectations and prior literature. While separation anxiety is typically associated with heightened dependency and fear of abandonment (Pini et al., 2021), some findings suggest that it may also be linked to ambivalent or distancing relational strategies under certain conditions (Finsaas and Klein, 2021; Cyranowski et al., 2002). Therefore, this result should be interpreted with caution. Several alternative explanations may be considered, including potential suppression effects among overlapping attachment dimensions (Cohen et al., 2013; MacKinnon et al., 2000), measurement characteristics of the ASAQ (Manicavasagar et al., 2003), and culturally shaped relational norms within the present sample (Markus and Kitayama, 2014). One possible interpretation is that individuals with higher levels of separation anxiety may engage in regulatory strategies that involve emotional distancing or withdrawal in order to manage overwhelming attachment-related distress, which in turn may reduce observable patterns of maladaptive dependency. However, given the cross-sectional design of the study, these interpretations remain tentative and require further investigation in longitudinal and culturally diverse samples.

Several alternative explanations may account for this unexpected negative association. First, statistical suppression may have occurred due to the strong shared variance among insecure attachment dimensions and separation anxiety. When highly overlapping predictors are included simultaneously, the unique effect of one variable may change direction once common variance is controlled (Cohen et al., 2013; MacKinnon et al., 2000). Second, the Adult Separation Anxiety Questionnaire primarily captures distress related to separation rather than compulsive relational engagement. Thus, individuals reporting high separation-related distress may also show withdrawal, ambivalence, or avoidance tendencies that reduce overt dependency behaviors (Manicavasagar et al., 2003). Third, cultural relational norms may shape how separation-related emotions are expressed. In collectivistic or family-oriented contexts, distress about separation may coexist with expectations of autonomy or relational restraint, potentially producing patterns that differ from those reported in Western samples (Markus and Kitayama, 2014). Taken together, these explanations suggest that the present finding should be interpreted cautiously and warrants replication in different cultural and methodological contexts.

At the same time, it is also plausible that separation anxiety may lead to heightened reassurance-seeking and clinging behaviors in some individuals, thereby increasing relational dependency rather than reducing it. The direction of this association may therefore depend on individual differences in attachment regulation strategies, such as hyperactivation versus deactivation. Future studies should examine whether these regulatory patterns moderate the relationship between separation anxiety and addiction-like relational dependency.

Cultural context may also be relevant in interpreting this pattern. In societies where family ties remain strong during emerging adulthood and relational autonomy develops gradually, separation-related distress may not necessarily translate into increased romantic dependency. Instead, individuals may experience tension between closeness expectations and norms of independence, which could manifest as emotional ambivalence rather than intensified relational involvement. This dynamic may be particularly relevant in the Turkish cultural context, where transitions to full relational independence often occur later and are embedded within ongoing family connectedness. Such cultural dynamics may help explain why separation-related concerns did not correspond to higher relational dependency in the present sample.

These results were consistent with expectations, in that anxious and avoidant dimensions of attachment style were significantly linked with increased levels of addiction-like dependency patterns in romantic relationships. These findings support attachment-based explanations of maladaptive relational dependency patterns in romantic relationships. Previous research has consistently shown that insecure attachment is associated with lower relationship satisfaction (Brauer and Proyer, 2020), reduced emotional security (Gori et al., 2023), and increased couple-related conflicts. From this perspective, attachment dimensions appear to capture core psychological processes underlying vulnerability to addiction-like relational dependency rather than establishing a distinct behavioral addiction construct.

The current study brings to the fore the great impact that a number of psychological factors can have on the issue of romantic relationship addiction. The core psychological factors that have been emphasized in the current study include anxious as well as avoidant attachment styles, mindfulness, as well as adult separation anxiety. It is evident that the manner in which individuals participate in romantic relationships can be inextricably tied to their attachment style as well as their mindfulness.

It is important to note that anxious and avoidant attachment styles could successfully predict romantic relationship addiction, thus validating the use of the attachment theory in this field of study. This finding supports other research that has found insecure attachment styles are related to decreased levels of satisfaction and security in romantic relationships.

Additionally, mindfulness was seen to exert a protective function through improved emotional regulation, empathy, and communication in romantic relationships. As supported in previous literature (Crane et al., 2017; Winter et al., 2021), mindfulness-based practices were related to better relationship harmony and conflict resolution. This further reinstates the relevance of mindfulness training in any psychoeducational intervention for addictive relationships.

At last, this research contributes to the increasing body of literature by combining attachment styles, mindfulness, and separation anxiety concepts in a unified model. Future studies conducted in this field may possibly provide added constructs or theoretical perspectives that may lead to the development of corresponding interventions for people at risk for maladaptive relationships.

### Limitations and future directions

Although this study makes several contributions, it also carries limitations that should be acknowledged. First, the cross-sectional design prevents causal interpretation of the associations among attachment, mindfulness, separation anxiety, and maladaptive relational dependency. Longitudinal research is needed to clarify temporal ordering among these variables. Second, the reliance on single-time self-report measures raises the possibility of response-set bias and common method variance, including social desirability effects. Future research should combine multiple data sources such as observational tasks, partner reports, or longitudinal designs to strengthen validity.

Another limitation concerns the operationalization of romantic relationship addiction. The present study relied on a codependency measure that captures maladaptive emotional reliance and relational enmeshment rather than behavioral addiction in a strict clinical sense, and this distinction should be considered when interpreting the findings. Although these constructs overlap conceptually, they are not fully equivalent, and future studies should employ alternative measures or latent modeling approaches to better differentiate relational dependency from insecure attachment and related constructs. The very strong bivariate association between anxious attachment and maladaptive relational dependency suggests substantial shared variance, and future studies should formally test discriminant validity using confirmatory factor analysis.

A further limitation relates to sample size justification. Although the obtained sample exceeded commonly recommended thresholds for SEM, a formal model-based power analysis was not conducted. Future research could employ Monte Carlo simulations or parameter-based power estimations to determine optimal sample sizes for testing complex relational models.

Finally, the sample consisted exclusively of individuals in emerging adulthood, which may limit generalizability to other developmental stages. Future studies should examine these relationships across broader age groups.

The unexpected negative association between separation anxiety and relational dependency should also be interpreted cautiously. Given the cross-sectional design and reliance on self-report measures, it is possible that shared variance among relational constructs, measurement characteristics of the separation anxiety scale, or statistical suppression effects influenced the direction of this association. Replication using longitudinal designs, alternative measures, and culturally diverse samples would help clarify the robustness of this finding.

### Data availability

Ethical issues and the matter of confidentiality in the study require that the datasets created and investigated here not be published. Requests to view the anonymized datasets can be made through the author.

## Conclusion

With the complexity observed in romantic relationships today, it is crucial to be able to identify the reasons behind the development of dependency in such associations. In exploring the subject matter, this particular study aims to establish links between psychological elements such as styles of attachment, mindfulness, and separation anxiety and their relation to addiction in romantic associations. Results revealed that there is greater vulnerability toward addiction-like relational dependency patterns for people who possess either anxious or avoidant styles of attachment in their romantic associations, whereas when it comes to their romantic experiences, their level of mindfulness shields them from such addiction patterns.

A rather unexpected finding is that relationship addiction is negatively associated with separation anxiety. Against received wisdom, higher levels of separation anxiety are actually associated with less addictive relationship behavior. This is an unexpected finding because, according to received wisdom, separation anxiety would be expected to increase relationship intimacy rather than reduce it.

In general, the findings highlight the importance of understanding the complex interaction between several skills of emotional regulation, attachment, and the personal strategies used to cope when experiencing a romantic relation addiction. This awareness of complexity may be crucial to successfully addressing interventions for people struggling with unhealthy forms of attachment in love.

### Recommendations

The findings appear to have a variety of applications, especially in encouraging healthier relationship patterns in young individuals. Several psychological issues relevant to interpersonal relations, including attachment, mindfulness, and separation-related coping, have been found to be successfully handled in either a preventative fashion or a therapeutic manner.

In order to enhance the development of more secure, satisfying, and healthy relationships, the following strategies are suggested:

Raise awareness about attachment patterns. Those with anxious or avoidant patterns of attachment may well benefit from involvement in group-based educational programs aimed at changing associated behaviors.

Practice mindfulness in romantic situations. Meditation, mindful breathing, and the examination of emotional triggers can aid an individual in maintaining a sense of objective reality and escaping dependent reactions to a romantic stimulus.

Offer organized support regarding separation distress. People exhibiting high levels of separation anxiety should have targeted interventions to maximize healthy adaptational strategies, considered to be between the extremes of interdependence and disconnection.

Additional training for mental health professionals: Mental health professionals, or therapists, that work with young adults could benefit from additional training that focuses on attachment-oriented, mindfulness-based interventions, specifically when dealing with romance-related issues or the symptoms of overdependence.

## Data Availability

The raw data supporting the conclusions of this article will be made available by the authors, without undue reservation.
